# Potent Acridone Antimalarial against All Three Life Stages of *Plasmodium*

**DOI:** 10.21203/rs.3.rs-6858328/v1

**Published:** 2025-06-27

**Authors:** Papireddy Kancharla, Rozalia A. Dodean, Yuexin Li, Xiaowei Zhang, Sean Kelly, Jordan Charlton, Angley Binauhan, Laurize Garcia, Diana Caridha, Michael S. Madejczyk, Xiannu Jin, William E. Dennis, Karl Kudyba, Sharon McEnearney, Partricia J. Lee, Cameron Blount, Jesse DeLuca, Chua Vuong, Kristina Pannone, Hieu T. Dinh, Kennedy Mdaki, Susan Leed, Monica L. Martin, Brandon S. Pybus, Anongruk Chimong, Liwang Cui, Joel Vega-Rodriguez, Martin Okitwi, Oriana Kreutzfeld, Christina Docan, Carol Green, Ying Liu, Aaron Agulay, Jon Mirsalis, Sarah N. Farrell, Geoffrey I. McFadden, Christopher D. Goodman, Alexandra S. Probst, Aaron Nilsen, Flaminia Catteruccia, Philip J. Rosenthal, Michael Riscoe, Roland Cooper, Alison Roth, Jane Kelly

**Affiliations:** 1 Department of Chemistry, Portland State University, Portland, Oregon 97201, United States; 2 Department of Veterans Affairs Medical Center, Portland, Oregon 97239, United States; 3 Department of Natural Sciences and Mathematics, Dominican University of California, San Rafael, California 94901, United States; 4 Experimental Therapeutics Branch, Walter Reed Army Institute of Research, Silver Spring, Maryland 20910, United States; 5 Department of Internal Medicine, Morsani College of Medicine, University of South Florida, Tampa, Florida 33612, United States; 6 Laboratory of Malaria and Vector Research, National Institute of Allergy and Infectious Diseases, National Institute of Health, Rockville, Maryland 20852, United States; 7 Infectious Diseases Research Collaboration, Kampala, Uganda; 8 Department of Medicine, University of California San Francisco, San Francisco, California 94143, United States; 9 SRI International, Biosciences Division, Menlo Park, California 94025, United States; 10 School of Biosciences, The University of Melbourne, Parkville, VIC, 3010, Australia; 11 Department of Immunology and Infectious Diseases, Harvard TH Chan School of Public Health, Boston, Massachusetts, 02115, United States; 12 Chemical Physiology and Biochemistry, Oregon Health & Science University, Portland, Oregon 97239, United States; 13 Howard Hughes Medical Institute, Boston, Massachusetts, 02115, United States; 14 Microbiology and Molecular Immunology, Oregon Health & Science University, Portland, Oregon 97239, United States

## Abstract

New antimalarial therapeutics ideally should target all three major *Plasmodium* life cycle stages. Here we present an acridone antimalarial chemotype that is potent against blood, liver, and mosquito stages of malaria parasites, with the potential for single-dose cure of bloodstream infections, radical cure of liver infections, and blocking of transmission to mosquitoes. Attributes of lead candidate T111 include potent in vitro activity against cultured parasites, ex vivo activity against clinical isolates, oral single dose cure in an asexual blood stage rodent model, inhibition of sexual blood stage parasites, activity against relapsing parasites in non-human primate liver cells, prevention of parasite development in mosquitoes, and synergy in combination with tafenoquine against blood and liver stage parasites. Analysis of parasites selected for resistance to T111 suggested inhibition of the mitochondrial electron transport chain, with a mechanism distinct from that of other antimalarials in use or under development. Safety profiles, including toxicology evaluations in rats, showed a favorable therapeutic index. Overall, T111 emerges as a promising candidate for treatment and prevention of malaria.

Malaria has plagued humans for thousands of years and remains one of the deadliest diseases in modern times, resulting in approximately a quarter billion clinical cases and over a half million deaths annually^1^. The most vulnerable group is children under the age of five, with the estimation that a child dies from malaria roughly every minute. *Plasmodium falciparum* is responsible for the majority of malaria mortality worldwide, with *P. vivax* the most widespread human malaria parasite, and other plasmodial species that infect humans each presenting different challenges^2^. Despite commendable progress in past decades, malaria control continues to face many challenges, including alarming resistance to artemisinin and some artemisinin-based combination therapy (ACT) partner drugs^3,4^, declining effectiveness of insecticides^5^, safety concerns associated with the limited radical cure options for relapsing *P. vivax and P. ovale* infections^6,7^, lack of transmission-blocking drugs^8^, and inability to provide full immunity with newly implemented vaccines^9^. Ideally, a chemotherapy approach to combat all stages and species of *Plasmodium* parasites would offer broad benefits toward the control and ultimate elimination of malaria^10,11^.

Malaria is a mosquito-borne infectious disease caused by *Plasmodium* parasites, with life cycles involving both vertebrate hosts and mosquito vectors^11–13^. Human infection begins when an infected female *Anopheles* mosquito bites a person, injecting sporozoites which enter the blood stream and invade liver cells, multiply asexually, and mature into schizonts. Thousands of merozoites are then released into the blood, where they invade red blood cells, multiply asexually, and cause clinical illness. Some blood stage parasites develop into sexual-stage gametocytes that are taken up by biting mosquitoes. Inside the mosquito, the parasites undergo sexual reproduction to develop into oocysts and then sporozoites. When the mosquito bites another human, sporozoites are injected, enabling continued transmission. Relapsing malaria, seen only with *P. vivax and P. ovale*, follows the reactivation of liver hypnozoites after extended periods of dormancy, leading to recurrent asexual bloodstream infection^14^.

Most of the antimalarial drugs currently in use target blood stage parasites, which are responsible for clinical manifestations^11,15^. The only available causal prophylactics that target liver stage infection are Malarone (atovaquone (ATV)-proguanil (PG)), primaquine (PQ), and newly approved tafenoquine (TQ). Of these, PQ and TQ are the only drugs that offer radical cures, killing hypnozoites to prevent relapsing *P. vivax* and *P. ovale* infections. In addition, low dose PQ is now recommended as a malaria transmission-blocking drug in some settings^7,16,17^. Unfortunately, PQ and TQ are both 8-aminoquinolines that cause hemolytic toxicity in patients with glucose-6-phophate dehydrogenase (G6PD) deficiency, a common enzyme abnormality^18,19^.

The advantages of antimalarial agents that kill malaria parasites in multiple life-cycle stages are self-evident, however the discovery of such highly desirable drugs remains an arduous challenge^11,20^. We have discovered and developed a novel acridone antimalarial chemotype, with T111 as the lead candidate, that exhibits potent activity against liver (including hypnozoite), blood (both asexual and sexual), and mosquito stage parasites, with single dose cure in a murine model as well as the potential to prevent relapsing infection and block transmission.

## RESULTS

### Antimalarial activity against blood stage *Plasmodium* parasites.

#### In vitro activity against asexual blood stage *P. falciparum*.

**Figure 1.**
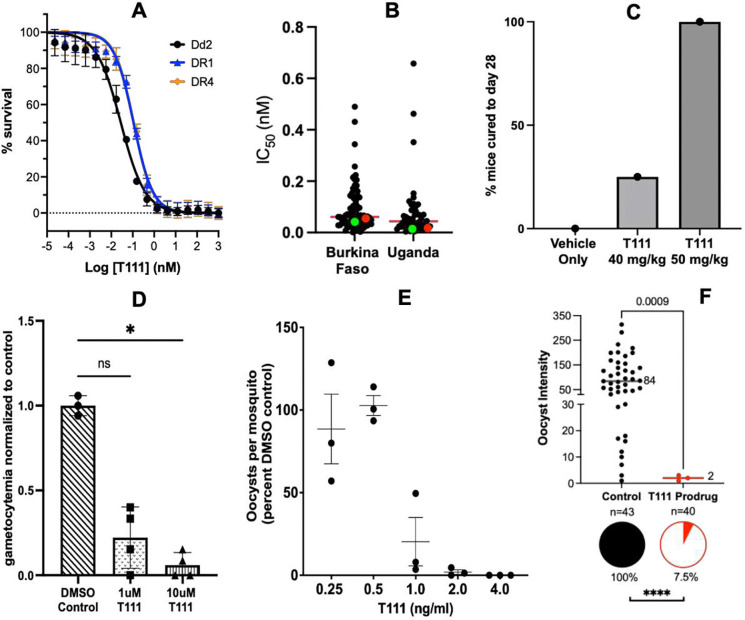
Antimalarial activities of T111 against blood and mosquito stage *Plasmodium* parasites. **A)** Dose response curves for activity against different asexual blood stage *P. falciparum* strains. *n* = 3, each performed in biological triplicates; error bars represent means of 3 independent experiments ± S.E. **B)** Ex vivo susceptibilities of blood stage *P. falciparum* clinical isolates from Burkina Faso and Uganda. Mean IC_50_ values are shown as red lines with means for control laboratory strains in red (3D7) and green (Dd2). **C)** In vivo efficacy in mice of a single oral dose of T111 against blood stage *P. yoelii*. **D)** In vitro inhibition of sexual blood stage *P. falciparum* gametocyte development. *n* = 2, each performed in duplicate;, one-way ANOVA with Kruskal-Wallis multiple comparison test, *P = 0.0163; error bars represent means ± S.D. **E)** In vivo inhibition of *P. falciparum* oocyst development in mosquitoes after direct membrane feeding. Values represent the means for 10–23 mosquitoes per treatment in each of 3 independent experiments. Oocyst numbers are normalized to the mean of untreated controls. Differences between treatments were significant at p = 0.011 (Friedman test). **F)** In vivo inhibition of *P. falciparum* oocyst development in mosquitoes after tarsal contact. Pie charts show prevalence of infection (proportion of mosquitoes with at least one oocyst; Fisher’s exact test, ****P<0.0001), and intensity graph shows the number of oocysts per infected mosquito (median lines indicated, Mann-Whitney test). n=number of mosquitoes, two biological replicates.

In our previous report^21^, T111 exhibited potent in vitro antiplasmodial activity against a panel of asexual blood stage *P. falciparum* parasites that included pan-sensitive strain D6, multi-drug resistant (MDR) Dd2, and ATV-resistant Tm90-C2B with sub- to low-nanomolar IC_50_ values. Considering the rising spread of artemisinin resistance^22^, T111 was also tested against the artemisinin-resistant *P. falciparum* clones DR1 and DR4 (Figure 1A), which were selected in the Dd2 strain with escalating dihydroartemisinin (DHA) pressure^23^. DHA had significantly decreased activity against these two resistant parasites (IC_50_ 89.5 nM for DR1 and 173.4 nM for DR4 compared to 8.30 nM for Dd2)^23^. T111 displayed excellent potency against Dd2 (IC_50_ = 0.028 nM), DR1 (IC_50_ = 0.11 nM), and DR4 (IC_50_ = 0.11 nM).

#### Ex vivo activity against asexual blood stage *P. falciparum* clinical isolates.

The activity of T111 against fresh *P. falciparum* isolates collected from patients diagnosed with falciparum malaria was evaluated in Burkina Faso and Uganda^24,25^. T111 demonstrated picomolar activity, with geometric mean IC_50_ values of 0.062 nM (0.0013 nM to 0.49 nM, N = 78) in Burkina Faso and 0.0044 nM (0.0037 nM to 0.66 nM, N = 50) in Uganda, with ex vivo activities similar to in vitro activities against the Dd2 and 3D7 control strains (Figure 1B).

#### In vivo oral efficacy against asexual blood stage *P. yoelii* in a rodent model.

To investigate oral efficacy against blood stage infections, we employed a well-established model using mice inoculated with *P. yoelii* (3.5 × 10^4^ per inoculum) via tail vein injection^26,27^. As previously reported by us^21^, T111 was curative when administered orally at 10 mg/kg/d once-daily for 4 days, beginning 24 hr post-inoculation. With interest in a single dose curative regimen, we also tested T111 administered orally once, 24 hr post-inoculation^28,29^. One of four mice treated with a single oral dose of 40 mg/kg, and all of four mice treated with 50 mg/kg were cured, with no parasites detected over 28 days (Figure 1C).

#### In vitro activity against sexual blood stage *P. falciparum* gametocytes.

The activity of T111 against sexual blood stage gametocytes was investigated after asexual stage *P. falciparum* NF54 parasites were stressed by starvation to induce gametocytogenesis^30,31^. The drug was added daily on days 5 to 7 post gametocyte induction, and parasitemia was determined on day 15, when control parasites had matured into late-stage gametocytes. T111 inhibited stage III and IV gametocytogenesis, with 75% and 93% suppression of gametocyte development at 1 μM and 10 μM, respectively (Figure 1D).

### Antimalarial activity against liver stage *Plasmodium* parasites.

#### In vitro activity against relapsing *Plasmodium cynomolgi* in rhesus hepatocytes.

*P. cynomolgi* is a malaria parasite of monkeys that is similar to *P. vivax*, including the risk of relapsing malaria due to emergence of liver hypnozoites. The antimalarial activity of T111 against relapsing liver stage malaria parasites was studied with a high-throughput in vitro assay using *P. cynomolgi* sporozoites and non-human primate (NHP) rhesus hepatocytes^32^, considering both prophylaxis (prevention of establishment of liver stage infection) and radical cure (elimination of liver schizonts and hypnozoites). T111 exhibited potent prophylactic activity against the development of liver stage hypnozoites, comparable or superior to that of the reference drugs TQ and ATV (Table 1). Remarkable prophylactic activity inhibiting schizont formation was also observed. Considering radical cure against developing schizonts and dormant hypnozoites, the potency of T111 was superior to those of reference drugs TQ and ATV for inhibition of both hypnozoites and schizonts. Cytotoxicity of T111 against simian hepatocytes was moderate (CC_50_ 0.67 uM), however, the cytotoxicity CC_50_ value vs human HepG2 hepatocyte cells was > 200 μM^21^.

### Antimalarial activity against mosquito stage *Plasmodium* parasites.

#### Standard membrane feeding assay (SMFA).

The transmission-blocking activity of T111 was investigated in a standard direct membrane feeding assay^33^, in which the drug was included during mosquito feeding with blood samples from a naturally infected *P. falciparum* NF54 strain gametocyte carrier. T111 inhibited oocyst development in a dose-dependent manner and eliminated oocyst formation at 4.0 ng/mL (9.5 nM) concentration (Figure 1E).

#### Tarsal contact assay.

In a tarsal contact assay^34^, female *Anopheles* mosquitoes were allowed to land on a thin film surface incorporated with a T111 prodrug at 5% by weight (structure shown in Scheme S1) for 6 min and one hour later fed a blood meal carrying *P. falciparum* NF54 gametocytes. Midguts of the infected mosquitoes were dissected on day 7. There was a significant, nearly complete reduction in oocyst prevalence (92.5% inhibition) and intensity (97.6% inhibition) in mosquitoes after brief tarsal contact with the T111 prodrug, whereas nearly all control mosquitoes showed a high intensity infection (Figure 1F).

### Synergy profiles of T111/TQ drug combination against blood and liver stage *Plasmodium* parasites.

#### In vitro synergy of the T111/TQ combination against asexual blood stage *P. falciparum* parasites.

**Figure 2.**
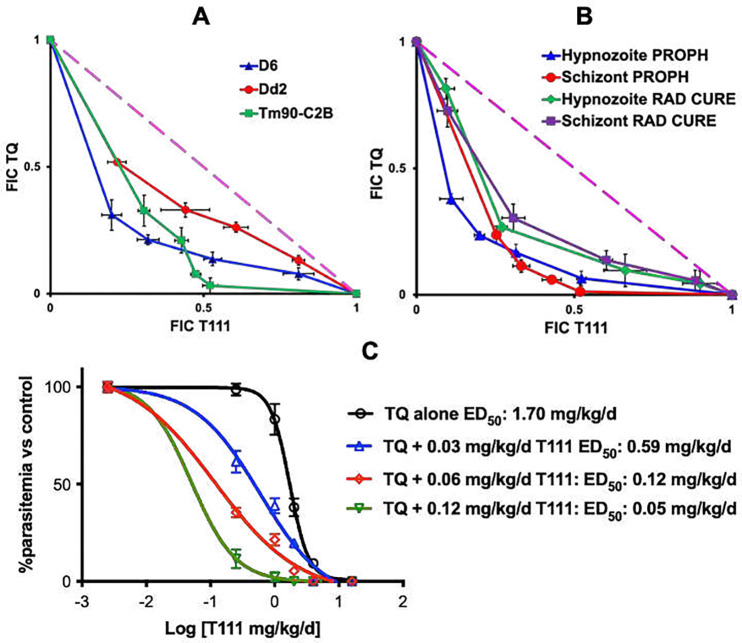
Drug-drug interactions between T111 and TQ against *Plasmodium* parasites. **A)** Isobologram for asexual blood stage *P. falciparum* parasites in vitro. **B)** Isobologram for liver stage rhesus *P. cynomolgi* with prophylactic (PROPH; drugs administered 1 hour post sporozoite infection on day 0, then continued on day 1 and 2) and radical cure (RAD CURE; drug exposure began on day 4, and continued on day 5, 6, and 7 post sporozoite infection) dosing. **C)** In vivo potentiation of TQ oral efficacy by T111 vs. *P. yoelii* in the blood stage 4-day suppression murine model.

Synergy was assessed by isobolar analysis with a well-established fixed ratio platform^35,36^, using a SYBR Green based assay^37^. As shown in Figure 2A, relative synergy between T111 and TQ was observed against pan-sensitive D6 (FIC index = 0.64), MDR Dd2 (FIC index = 0.82), and ATV-resistant Tm90-C2B (FIC index = 0.59).

#### In vitro synergy of the T111/TQ combination against relapsing *P. cynomolgi* parasites.

Using the NHP *P. cynomolgi* assay described above^32^, for prophylaxis, synergy was evident in the inhibition of both hypnozoite and schizont development, with FIC index values for the TQ/T111 combination at 0.49 and 0.48, respectively. For radical cure, synergy was also observed in inhibiting hypnozoite and schizont development, with FIC index values at 0.78 and 0.77, respectively (Figure 2B).

#### In vivo potentiation of TQ against blood stage *P. yoelii* in the 4-day suppression murine model.

We compared activities of TQ alone and in the presence of sub-therapeutic oral dosages of T111 in the 4-day suppression model. T111 potentiated the oral efficacy of TQ, with a 34-fold reduction in ED_50_ values at 0.12 mg/kg/d (Figure 2C).

#### In vivo single dose cure synergy of the T111/TQ combination against *P. yoelii* in mice.

We also investigated the combination of T111 and TQ as a single dose cure in the *P. yoelii* model. TQ achieved single dose cure in all treated mice at 40 mg/kg (Table 2). T111 achieved single dose cure in all treated mice at 50 mg/kg. Various lower dosage combinations of TQ with T111 achieved single dose cures (Table 2).

#### In vivo synergy of the T111/TQ combination against liver stage *P. berghei* in mice.

**Figure 3.**
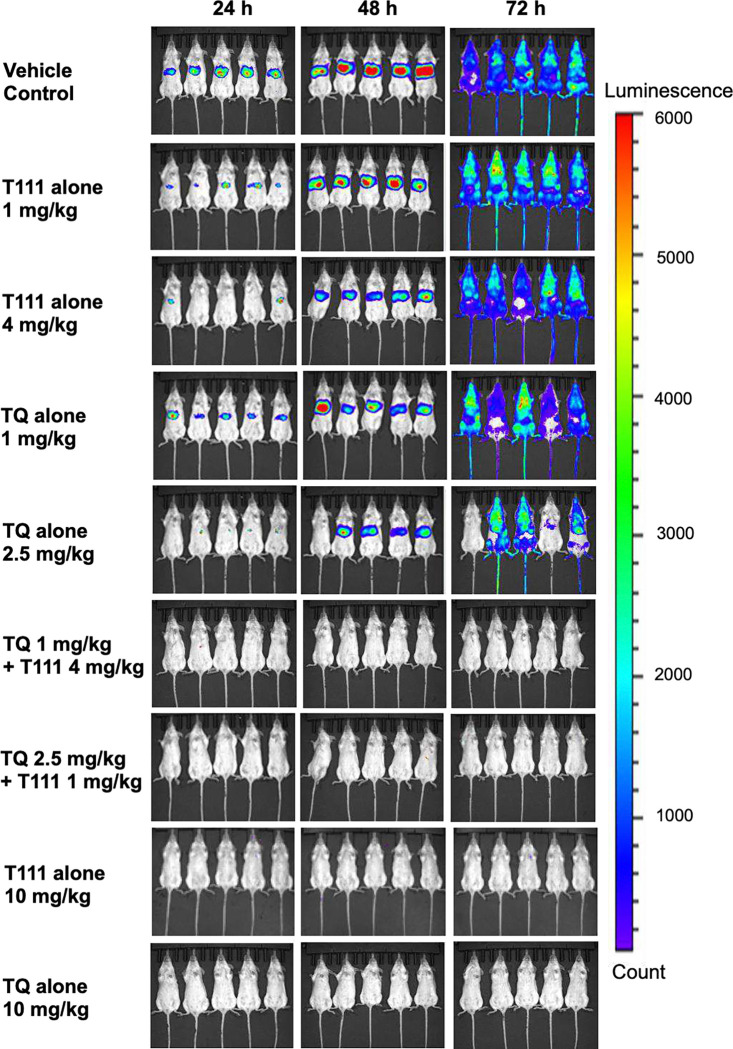
Bioluminescence and real time in vivo imaging of parasite loads in mice with or without treatment of test drugs or test drug combinations. The bioluminescence level represents the parasite burden. Intensity of bioluminescence is indicated by the color scale.

We previously reported in vivo oral efficacy of T111 against liver stage *Plasmodium* infection^21^. A real-time in vivo imaging system (IVIS) using transgenic bioluminescent parasites to quantify parasite development was employed to study liver stage infection in live anesthetized *P. berghei* infected mice^26,38,39^. Potential synergy between T111 and TQ was investigated using the same model. Luciferase-expressing *P. berghei* sporozoites were inoculated in mice on day 0, with oral doses of drugs administered on days −1, 0, and 1 and bioluminescence signals were measured at 24 and 48 hr after inoculation for liver stage development and at 72 hr for blood stage infection in anesthetized mice. Strong bioluminescence signals were detected in untreated mice at both 24 and 48 hr in the liver, followed by intense whole-body signals at 72 hr (Figure 3). Treatment with TQ at 1 mg/kg/d or 2.5 mg/kg/d, and with T111 at 1 mg/kg/d or 4 mg/kg/d did not clear liver stage infection, and all treated mice developed blood stage infection. Treatment with TQ at 10 mg/kg/d or T111 at 10 mg/kg/d provided full protection from liver stage infections. Treatment with the combination of 1 mg/kg/d TQ + 4 mg/kg/d T111 provided full protection, without visible parasites in the liver at 24 and 48 hr or the blood over 31 days after inoculation. The same results were observed with the combination of 2.5 mg/kg/d TQ + 1 mg/kg/d T111.

### Safety and toxicology evaluation.

#### In vitro safety evaluations.

**Figure 4.**
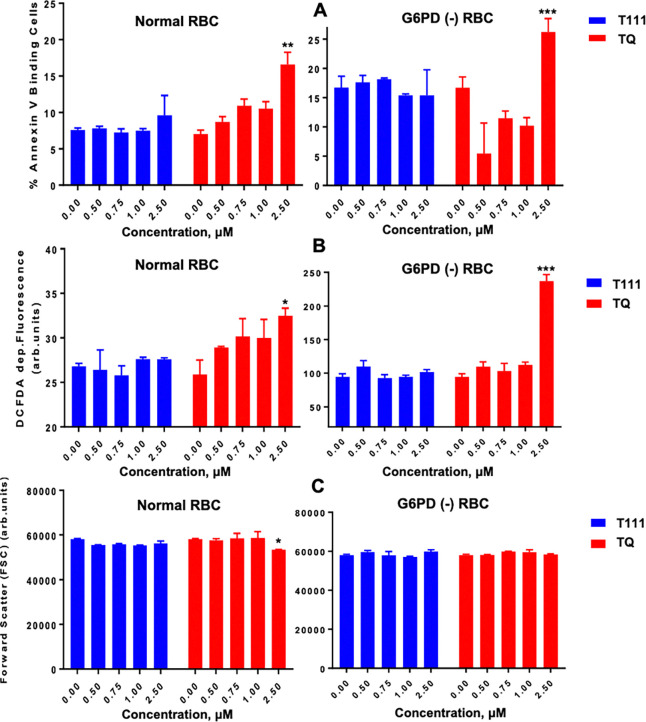
Effect of T111 and TQ on eryptosis. **A)** Phosphatidylserine exposure, shown as RBC annexin V binding after 48 hr incubation with drugs. **B)** Reactive oxygen species formation, shown as 2’,7’-dichlorodihydrofluorescenin diacetate (DCFDA) fluorescence after 48 hr exposure to drugs. **C)** The size/volume of RBC, shown as forward scatter after 48 hr exposure to drugs. Two-way ANOVA comparison test. *p < 0.05, **p < 0.005, ***p < 0.0005.

Assessment of cytotoxicity against human hepatic HepG2 cells using the MTT assay^40^ showed no apparent toxicity, with CC_50_ > 200 μM, indicating a selective index > 10,000. hERG channel inhibition assessment using an automatic parallel patch clamp system^41^ showed low risk of cardiac toxicity, with IC_50_ > 100 μM (Supplementary Table S1). The Ames test against *Salmonella typhimurium* TA98 and TA100 strains with and without S9 activation^42^ showed a negative result at up to 10 μM, without any increase over the background reversion rate, indicating a low risk of mutagenicity (Supplementary Figure S1). Evaluation of eryptosis as a marker for potential drug induced hemolytic toxicity, characterized by cell shrinkage, oxidative stress, and phosphatidylserine translocation to cell surface^43^, did not show the toxicity seen with TQ against G6PD-deficient RBCs (Figure 4).

#### In vivo toxicology and toxicokinetic (TK) analysis in rats.

Murine blood stage and liver stage efficacy studies included observations of animal weight, grooming, posture, and locomotion. No overt clinical toxicity or behavior change was observed in any T111 treated mice (highest tested dose: 30 mg/kg/d × 4 days and 80 mg/kg × 1 day). In addition, extensive safety evaluation of T111 was conducted in rats, with both single dose and repeat dose toxicology studies. In the maximum tolerated dose (MTD) determination study, a single oral dose of T111 was administered by gavage at 30, 100, 200, and 400 mg/kg to male and female Sprague Dawley rats, and animals were observed for two days. Clinical observations were performed immediately, 2–4 hr and 24 hr post dosing. T111 was well tolerated, with no clinical signs suggesting toxicity. A single-dose MTD could not be established due to limited solubility of T111 in the vehicle (PEG-400), but it is considered to be greater than 400 mg/kg, the highest dose tested. Based on the observed effects and T111 solubility limitations in the MTD studies, the dose levels selected for 7-day repeat dose studies were 25, 100 and 400 mg/kg. Male and female Sprague Dawley rats were given T111 daily for 7 consecutive days via oral gavage then euthanized and evaluated for body weight changes, clinical signs, plasma drug concentrations, organ weights, gross and microscopic examinations of selected tissues, and clinical pathology (hematology, clinical chemistry). These repeated daily oral administrations were well tolerated, albeit with minor clinical signs, specifically, shoveling and ruffled fur in T111-treated animals. Repetitive motions were observed in T111-treated females at 100 mg/kg and 400 mg/kg and in one control male and one treated male in the 400 mg/kg treatment group. Pink discharge in the eye and/or nostril areas (chromodacryorrhea) was observed with increased frequency in T111-treated females relative to controls, as well as two males from the 25 mg/kg treatment group and one male from the 100 mg/kg treatment group. No significant alterations in body weight, organ weight, clinical pathology, or histopathology were observed. For TK analysis, T111 plasma levels were determined at 6 time points on Day 1 and Day 7 in male and female rats. T111 was not completely cleared within 24 hr, indicating potential accumulation upon repeated daily dosing (Table 3). Maximum systemic exposure was obtained in the 100 mg/kg dose group, likely due to the limited solubility at 400 mg/kg, which was administered as a suspension (the lower doses were administered as solutions).. The TK profile of T111 in these studies was consistent with our previously published PK parameters in mice, which indicated a long half-life and rapid absorption^21^.

### Mechanistic studies for target identification and validation.

#### Drug resistance selection.

**Figure 5.**
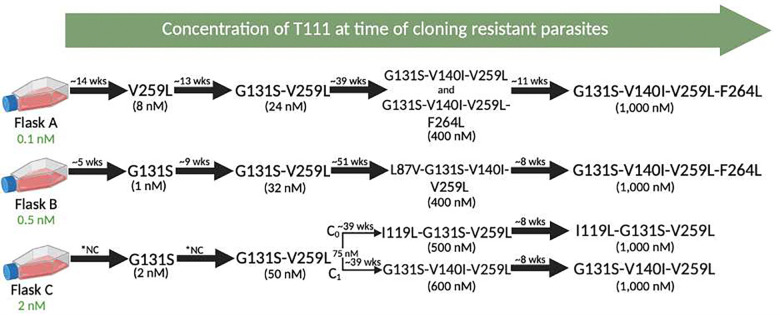
Selection of cyt b mutations in cultures incubated with the indicated concentrations of T111 for the indicated durations. *NC: not calculated; wks: weeks.

To gain insight into the mechanism of action and propensity for drug resistance selection, we cultured *P. falciparum* Dd2 strain under continuous and incrementally increasing concentrations of T111. Over 16–18 months, the parasites acquired resistance to T111 in an ordered and sequential manner (Figure 5). Considering that earlier generations of our acridone chemotype had a presumed target of *Plasmodium* cyt *b*^44^, we sequenced *pfcytb* in parasites with decreased susceptibility to T111. Multiple mutations were identified in the cyt *b* gene in the resistant parasites (Figure 5). Initial selection led to modest resistance in parasites with the V259L or G131S mutation. Continued selection yielded parasites with the combination of these two mutations, and subsequent selection with high nanomolar concentrations of T111 yielded parasites with 3 or 4 *pfcytb* mutations, including two novel mutations V140I and I119L. All mutations selected, except, L87V, are predicted to be located in the cyt *b* Q_o_ site^45^.

To confirm that cyt *b* mutant haplotypes confer T111 resistance, we tested susceptibility of the mutant lines to T111 and other compounds known to target the mitochondrial electron transport chain (ETC) (Table 4). T111 retained low nanomolar potency against parasites with two cyt *b* mutations, but a third mutation conferred high level resistance. T111-resistant mutant lines did not show significant cross resistance with ATV (Q_o_ site inhibitor), but had various degrees of cross resistance with other Q_o_ site inhibitors (ELQ-400 and MYX). Two T111-resistant mutant lines, Dd2-C0I119L-G131S-V259L and Dd2-C1G131S-V140I-V259L, exhibited significant cross resistance with ELQ-300 (Q_i_ site inhibitor) and DSM265 (DHODH inhibitor), and sequencing revealed that they also carried the *pfdhodh* C276F mutation. There was no cross resistance with the clinically deployed antimalarials chloroquine, piperaquine, lumefantrine, or artemisinin derivatives (Table S2 in Supplementary Information), which do not act against the ETC.

#### Cross-resistance profile with *Plasmodium* mitochondrial electron transport chain (ETC) inhibitors with different site preference.

To study cross-resistance between T111 and other ETC inhibitors, we used a panel of *P. falciparum* parasites with different cyt *b* Q_o_/Q_i_ site sequences (Table 5). Clinical isolate Tm90-C2B carries a point mutation at the Q_o_ site of cyt *b* (Y268S) that confers ATV resistance^46^. The Dd2-D_1_^I22L^ clone was selected by culturing *P. falciparum* Dd2 under ELQ-300 drug pressure, leading to an I22L mutation in the Q_i_ region of cyt *b* contributing to loss of sensitivity to ELQ-300^47^. *P. falciparum* transgenic D10yDHOD parasites, which bypass the parasite’s ETC fueled pyrimidine synthesis pathway, are therefore completely resistant to all mitochondrial ETC inhibitors^48,49^. We also included pan-sensitive (D6) and MDR (Dd2) strains of *P. falciparum* as reference parasites. T111 exhibited decreased activity against ATV-resistant parasites, but retained nanomolar potency (IC_50_ vs Tm90-C2B = 5.6 nM). Interestingly, T111 had enhanced activity against parasites resistant to the Q_i_ site inhibitor ELQ-300 (IC_50_ of T111 vs Dd2-D_1_^I22L^ = 0.0008 nM). In contrast, ATV and ELQ-300 had decreased activity against Tm90-C2B (ATV IC_50_ = 8,256 nM) and Dd2-D_1_^I22L^ (ELQ-300 IC_50_ = 224 nM), respectively. Similar to the ETC inhibitors ATV, ELQ-300, and DSM265, T111 had reduced activity against D10yDHOD, with a resistance index >1000, confirming that T111 targets the mitochondrial ETC.

## DISCUSSION

Significant new findings have emerged since the initial discovery of the antimalarial acridone chemotype^21,44^, leading to the development of a preclinical candidate (T111) with the following attributes: 1) in vitro inhibition of *P. falciparum* asexual blood stage growth at picomolar concentrations with a highly favorable selectivity index^21^; 2) ex vivo picomolar activity against fresh isolates from *P. falciparum* infected African patients; 3) in vitro inhibition of sexual blood stage *P. falciparum* gametocyte formation; 4) in vivo oral curative efficacy (including single dose cure) in an erythrocytic *P. yoelii* murine model; 5) in vitro prevention of *P. berghei* development in human hepatocytes at low nanomolar concentrations^21^; 6) in vitro prevention and inhibition of relapsing *P. cynomolgi* hypnozoite and schizont development in rhesus hepatocytes at nanomolar concentrations; 7) in vivo protection from *P. berghei* liver infection and cure of blood infection via oral administration in mice^21^; 8) in vitro synergy with TQ against erythrocytic *P. falciparum*; 9) in vitro synergy with TQ against relapsing *P. cynomolgi* in rhesus hepatocytes; 10) in vivo potentiation of TQ oral efficacy against blood stage *P. yoelii* murine malaria; 11) in vivo synergy with TQ against liver stage *P. berghei* infection in mice; 12) in vivo inhibition of *P. berghei* and *P. falciparum* sporozoite formation in mosquitoes^50^; 13) in vivo prevention of oocyst formation in mosquitoes at nanomolar concentrations; 14) in vivo disruption of oocyst formation in mosquitoes by tarsal contact; 15) promising safety profiles, with low in vitro hERG inhibition, a negative Ames result, and no observed T111 induced eryptosis in both normal and G6PD deficient red blood cells; 16) good tolerability in animals without observed toxicity; and 17) feasible DMPK profiles, with rapid absorption, a long half-life, and high drug concentrations in the liver^21^. Furthermore, an efficient four-step synthesis from commercially available starting materials enables cost-effective large-scale production of T111.

Extensive safety profiling of T111 indicated lack of hemolytic toxicity or toxicological findings in experimental animals. We demonstrated potency of T111 against liver stage *Plasmodium* hypnozoites and strong synergy with TQ in preventing liver stage infection (including hypnozoites) and treating blood stage infection (including single dose cure) using multiple in vitro and animal model assays. These findings suggest that the addition of T111 allows dose reduction for TQ which might mitigate toxicity. Given the paucity of hypnozoitocidal candidates and narrow safety profiles with TQ, the radical cure activity of T111 is promising, whether on its own or as a synergistic partner to improve the TQ therapeutic window for treatment and prevention of vivax malaria, including in G6PD deficient patients. Further testing of the anti-relapse efficacy of T111 and its potential synergy with TQ in a NHP model is underway.

Previously, the Goodman group reported that T111 completely inhibited sporozoite development when drugs were fed to infected mosquitoes, with potency against both *P. berghei* and *P. falciparum* superior to that of ATV and all other tested drugs (either in clinical use or under development)^50^. Here we report that T111 completely prevents *P. falciparum* oocyst formation in a direct membrane feeding assay that simulates a blood meal taken from a treated human and significantly disrupts oocyst formation in a tarsal contact assay that mimics uptake of the drug after landing on an antimalarial infused bed net. These findings offer direct evidence of transmission blocking potential for T111, establishing a new potential role for this antimalarial chemotype.

The *Plasmodium* mitochondrial ETC, including the cytochrome *bc*_*1*_ (cyt *bc*_1_) complex and DHODH, is essential for parasite survival in human and mosquito stages, and a validated drug target^51^. The Q cycle inside the cyt *bc*_1_ complex has two quinone-binding sites, the quinol oxidation site (Q_o_) and quinone reduction site (Q_i_), located on opposite sides of the membrane and linked by a transmembrane electron-transfer chain. ATV is the only antimalarial ETC inhibitor in clinical use, and it targets the Q_o_ site, with resistance associated with point mutations at this site^46^. Selection with T111 yielded resistance associated with the sequential acquisition of mutations in the Q_o_ site. However, there are important distinctions between T111 and ATV, both genetically and functionally. First, T111 is highly refractory to resistance selection as parasites required 16–18 months of constant selection pressure to become highly resistant to T111, whereas ATV resistance can be easily generated in a few weeks^52,53^. Second, T111 selection did not generate the clinically important Y268S mutation that renders ATV inactive. T111 retained nanomolar potency against parasites with two cyt *b* mutations, and the selection of a third mutation was necessary to confer high level resistance. Third, T111 remained highly potent against ATV-resistant parasites, and reciprocally, T111-resistant parasites did not show significant cross resistance with ATV. Unlike T111, ATV is inactive against *Plasmodium* liver stage hypnozoites^54^ and sexual blood stage gametocytes^55^. Extensive cross-resistance profiling using parasites with various resistance profiles and ETC inhibitors with different Q_o_/Q_i_ site preferences demonstrated that T111 had picomolar to nanomolar potency against parasites resistant to other Q_o_ or Q_i_ site inhibitors. These mechanistic investigations suggest a complex mode of action for T111. *In vitro* selected T111 resistance was enhanced by a mutation in *pfdhodh*, suggesting that T111 interactions extend beyond the Q_o_ site of the ETC. Targeting subtle interactions within the ETC that are crucial to antimalarial potency and resistance has allowed us to overcome ATV resistance, adding support for the ETC as an important target for new antimalarials. Development of new ETC inhibitors is further encouraged by the inability of ATV resistant parasites to survive in mosquitoes, blocking the spread of resistance^33,56^.

We have developed a novel antimalarial chemotype that has potent activity against the main life cycle stages of *Plasmodium* parasites, with our lead candidate (T111) meeting all five current target candidate profiles proposed by Medicines for Malaria Venture: molecules that clear asexual blood-stage parasitemia (TCP-1); are active against hypnozoites (TCP-3); are active against hepatic schizonts (TCP-4); that block transmission by targeting parasite gametocytes (TCP-5); and that block transmission by targeting the insect vector (TCP-6)^11,16^. In addition, T111 exhibited strong synergistic interaction with TQ against both liver and blood stage parasites, excellent safety profiles, and optimal DMPK properties, with a suggested mechanism of action distinct from that of other antimalarials currently in use or under development. The combination of these features in T111 is unique and promising, potentially offering a new drug with distinctive properties for antimalarial treatment and chemoprevention.

## Methods

### Synthesis of T111 and T111 prodrug.

The original synthetic method for preparing T111 was described in our previous publication^21^. The revised synthetic approach for scale-up production of T111, as well as the synthetic method for its prodrug, are provided in the Supplementary Information. Synthetic method for the preparation of T111 prodrug is also provided in Supplementary Information.

### In vitro drug susceptibility assay against ART-resistant parasites.

Drug susceptibility assay against ART-resistant *P. falciparum* clones was performed as previously described using a SYBR Green based assay^37,57^. Additional descriptions of the method are provided in Supplementary Information.

### Ex vivo drug susceptibility assay against clinical isolates.

The ex vivo antiplasmodial activity of T111 was tested against fresh *P. falciparum* isolates using a 72-hr growth inhibition assay with SYBR Green detection, as described in previous publications^24,25^. Isolates were collected from patients with uncomplicated falciparum malaria in Bobo-Dioulasso, Burkina Faso and Tororo, Uganda. Additional descriptions of the methods are provided in Supplementary Information.

### In vivo antimalarial efficacy against blood stage *P. yoelii* in rodent model.

The efficacy of T111 and the T111/TQ combination was determined using two well-established murine models against *P. yoelii* parasites: 1) a 4-day suppression model^21,44,58,59^; and 2) a single dose cure model^28,29,59^. Additional descriptions of the method are provided in Supplementary Information.

### Animals and Ethical Statement at Portland VA Medical Center (PVAMC).

In vivo blood stage antimalarial testing was carried out at PVAMC under protocols approved by the PVAMC IACUC. Research was conducted in an AAALACi (Association for Assessment and Accreditation of Laboratory Animal Care International)-accredited facility in compliance with the Animal Welfare Act and other federal statutes and regulations relating to animals and experiments involving animals adhering to principles stated in the *Guide for the Care and Use of Laboratory Animals* (NRC Publication Eighth edition)^60^.

### In vitro antiplasmodial activity against sexual blood stage *P. falciparum* gametocytes.

*P. falciparum* NF54 was used to generate gametocytes. The erythrocytic stage of the parasite was cultured following standard laboratory protocols. Gametocyte production was carried out as described by Tripathi et al.^31^ Additional descriptions of the method are provided in Supplementary Information.

### In vitro liver stage *P. cynomolgi* assay.

As described previously, the liver stage *P. cynomolgi* assay is designed to identify anti-hypnozoite drugs utilizing a 384-well in vitro culture system^32^. Detailed method was described in our recent publication^59^ and additional descriptions of the method are provided in Supplementary Information.

### Ethical Statement.

*Anopheles dirus* mosquitoes infected with the B strain of *P. cynomolgi* were produced by the Department of Entomology at the Armed Forces Research Institute of Medical Sciences (AFRIMS; Bangkok, Thailand) under an IACUC approved animal use protocol. The USAMD-AFRIMS IACUC Review Division, U.S. Army Medical Research and Materiel Command, reviewed and approved this study. Animals were maintained in accordance with established principles under the Guide for the Care and Use of Laboratory Animals and the Animals for Scientific Purposes Act and its subsequent regulations. The USAMD-AFRIMS animal care and use program is fully accredited by the AAALACi.

### In vivo antimalarial efficacy against liver stage *P. berghei* in rodent model.

IVIS system was used to determine the in vivo liver stage efficacy of T111 or the T111/TQ combinations in anesthetized mice, as previously described^21,38,39,44,59^. Additional descriptions of the method are provided in Supplementary Information.

### Animals and Ethical Statements at Walter Reed Army Institute of Research (WRAIR).

Four to five weeks old male ICR-CD1 mice (Charles River Laboratories. Inc. Raleigh, NC) weighing 23–35 g were used for PK studies. Four- to six-week-old female Albino C47BL/6 mice weighing 18–22 g (Jackson Laboratories, Bar Harbor, ME) were used to conduct LS in vivo efficacy studies. The mice were left to acclimatize for 7 days prior to the beginning of research studies. All animals were assigned a study number with an individual ear tag. Cards attached to each animal cage were also used to identify the study groups. All animals were quarantined for stabilization for 7 days prior to infection. Mice were housed in a designated room with food and water supplied ad libitum and in 12:12 light/dark cycle.

The animal protocol for this study was approved by the Walter Reed Army Institute of Research, Institutional Animal Care and Use Committee in accordance with national and Department of Defense guidelines. Research was conducted in an AAALACi accredited facility in compliance with the Animal Welfare Act and other federal statutes and regulations relating to animals and experiments involving animals and adheres to principles stated in the Guide for the Care and Use of Laboratory Animals, NRC Publication, 2011 edition^60^.

### Membrane feeding assay (SMFA) in mosquitoes.

The SMFA assay was performed using *P. falciparum* NF54 gametocytes produced in vitro as described previously^33^. Additional descriptions of the method are provided in Supplementary Information.

### Tarsal contact assay in mosquitoes.

The tarsal assay using a thin film was performed as previously described^34^. The method for preparation of T111 prodrug and thin film, as well as additional descriptions of the tarsal assay method are provided in Supplementary Information.

### In vitro drug combination studies against blood stage *P. falciparum* and liver stage *P. cynomolgi*.

For in vitro T111/TQ drug combination studies, blood stage and liver stage drug susceptibility tests were performed as described above, using a fixed-ratio platform for drug combinations, as previously described^35,36,61^. Additional descriptions of the method are provided in Supplementary Information.

### In vivo drug combination studies against blood stage *P. yoelii* and liver stage *P. berghei* in mice.

In vivo antimalarial efficacy studies were performed as described above. In the blood stage 4-day suppression model, the effect of T111 on TQ oral efficacy was conducted by comparing dose-response curves of TQ alone and in the presence of sub-therapeutic oral doses of T111, and the potentiation effect was expressed as the ratio of ED_50_ values for TQ and in the presence of T111. In the blood stage single dose cure model, various oral dose combinations of T111 with TQ were administered in mice 24 hr post infection, and corresponding curative effects (no parasites detected) were determined on day 28. For liver stage in vivo oral efficacy studies, T111 alone, TQ alone, and sub-therapeutic combinations were administered to mice days −1, 0, +1 after parasite inoculation. Parasite loads in the liver at 24 and 48 hr were determined for liver stage protection, and blood stage cure was determined on day 31.

### hERG channel inhibition assay.

The hERG assay was performed at Eurofins Panlabs Inc. St Charles, MO, United States. T111 was tested using the standard hERG potassium channel assay^41^ at six concentrations from a top concentration of 100 *μ*M. Verapamil was used a positive control.

### Ames test.

Mutagenicity evaluation was assessed using the Ames assay^42^ (EBPI, Ontario, Canada) against *S. typhimurium* TA100 and TA98, with and without S9 activation. Experimental procedures were described in previous publications and additional descriptions of the method are provided in Supplementary Information.

### Eryptosis assay.

In vitro evaluation of eryptosis as a marker for induced hemolytic toxicity was performed using deidentified human blood samples obtained from the Clinical Trial Center at WRAIR, accordingly to previously described methods^43,62,63^. The study was approved by the ethics committee of the WRAIR. Details are provided in Supplementary Information.

### In vivo toxicology and toxicokinetic studies in rats.

In vivo toxicology and toxicokinetic studies in rats were performed at SRI International, using the methods previously described. Detailed methods are provided in Supplementary Information.

### Drug resistance selection studies.

Drug resistance selection studies were performed by culturing *P. falciparum* Dd2 parasite under incremental and continuous drug pressure of T111, using the method similar to those previously described^44,64^. Details are provided in Supplementary Information.

### Drug subspeciality testing for cross-resistance profile.

Drug susceptibility assays against *P. falciparum* strains were performed using a SYBR Green based assay, as previously described^37^. D6 and Dd2 were obtained from MR4. Tm90-C2B was obtained from WRAIR. Dd2-D_1_^I22L^ and ELQ-300 were obtained from Portland VA Medical Center. D10yDHOD was obtained as a courtesy from Dr. Akhil Vaidya at Drexel University.

## Supplementary Material

Supplementary Files

This is a list of supplementary files associated with this preprint. Click to download.

SupplementaryInformation.pdf

## Figures and Tables

**Table 1.** In vitro antiplasmodial activity against liver stage *P. cynomolgi* in NHP hepatocytes.

drug	inhibition IC_50_ (μM)^a^ vs NHP *P. cynomolgi*				cytotoxicity CC_50_ (μM)^a^ vs simian hepatocytes

	prophylactic mode^b^		radical cure mode^c^		

	hypnozoite	schizont	hypnozoite	schizont	

T111	0.098±0.033	0.014±0.0050	0.35±0.035	0.24±0.083	0.67±0.24
TQ	0.24±0.035	0.12±0.028	8.48±0.98	10.92±1.56	15.2±2.32
ATV	0.39±0.43	0.008±0.003	2.45±0.30	2.27±0.38	2.01±0.14
MAD	0.025±0.0070	0.023±0.0070	0.028±0.0070	0.036±0.015	4.67±2.03

a IC_50_ and CC_50_ values are the mean ± SEM from 4 independent experiments, each in quadruplicate

b drugs were administered 1 hour post sporozoite infection on day 0

c drug exposure began on day 4, and continued on day 5, 6, and 7 post sporozoite infection

TQ: tafenoquine; ATV: atovaquone; MAD: maduramicin.

**Table 2.** The effect of T111 on TQ in the single dose cure rodent *P. yoelii* model.

drug combinations (mg/kg)		number of mice cured (% survival)

Tafenoquine	T111	

15	0	0/4 (0%)
20	0	1/4 (25%)
30	0	3/4 (75%)
40	0	4/4 (100%)
0	20	0/4 (0%)
0	30	0/4 (0%)
0	40	1/4 (25%)
0	50	4/4 (100%)
5	5	0/4 (0%)
5	10	0/4 (0%)
5	15	3/4 (75%)
5	20	4/4 (100%)
10	5	0/4 (100%)
10	10	3/4 (75%)
10	15	4/4 (100%)
10	20	4/4 (100%)
15	5	4/4 (100%)
15	10	4/4 (100%)
15	15	4/4 (100%)
15	20	4/4 (100%)
20	5	4/4 (100%)
20	10	4/4 (100%)
20	15	4/4 (100%)
20	20	4/4 (100%)

**Table 3.** Plasma TK parameters of T111 in male and female rats after single oral administration as single dose (Day 1) or after seven daily consecutive doses (Day 7).

	Day 1						Day 7					
	
drug dose (mg/kg)	male			female			male			female		

	25	100	400	25	100	400	25	100	400	25	100	400
	
*t_1/2_* (hr)	*NC	*NC	*NC	*NC	*NC	*NC	21	25	18	13.5	14.5	15
T_max_ (hr)	8	8	8	8	24	1	8	2	8	8	2	8
C_max_ (ng/mL)	499	592	367	439	532	365	499	676	507	338	1,070	847
AUC_last_ (hr.ng/mL)	9,300	11,100	5,340	8,090	10,700	4,400	9,570	17,700	14,900	11,100	29,900	16,500
AUC_inf_ (hr.ng/mL)	*NC	*NC	*NC	*NC	*NC	*NC	6,400	19,100	15,000	11,100	30,300	16,600
Cl/F (mL/hr/kg)	*NC	*NC	*NC	*NC	*NC	*NC	2,780	5,960	33,300	2,870	4,260	31,600

* NC: not calculated. t_1/2_, AUC_inf_, and Cl/F on Day 1 could not be calculated due to insufficient data points in the terminal phase of the plasma profile.

**Table 4.** Antiplasmodial activity of ETC inhibitors against *P. falciparum* T111 mutants.

Parasite Line	Inhibition IC_50_ vs. *P. falciparum* (nM)*					

	T111	MYX	ATV	ELQ400	ELQ300	DSM265

Dd2	0.1 ± 0.02	24 ± 3.6	0.4 ± 0.05	4.0 ± 0.5	38 ± 5.0	3.2 ± 0.3
Dd2^131S^	0.8 ± 0.08	383 ± 38^c^	0.3 ± 0.05	4.2 ± 0.4	29 ± 5.8	1.9 ± 0.2
Dd2^259L^	4.5 ± 0.7	64 ± 13	6.6 ± 1.9	24 ± 6.7	22 ± 6.4	3.3 ± 1.0
Dd2^131S-259L^	12 ± 2.5	975 ± 139^a^	1.5 ± 0.2	28 ± 13	24 ± 3.5	2.8 ± 1.2
Dd2-A^131S-140I-259L^	74 ± 5.1^a^	192 ± 11	4.7 ± 0.4	133 ± 17	52 ± 5.5	3.2 ± 0.5
Dd2-A^131S-140I-259L-264L^	98 ± 12^a^	16 ± 4.1	15 ± 2.0^a^	80 ± 15	35 ± 3.2	1.2 ± 0.07
Dd2-B^87V-131S-140I-259L^	129 ± 6.3^a^	373 ± 11^c^	10 ± 1.4^c^	177 ± 3.1^d^	67 ± 4.6	3.5 ± 0.4
Dd2-B^131S-140I-259L-264L^	114 ± 13^a^	34 ± 3.3	11 ± 1.8^c^	126 ± 32	25 ± 2.7	2.8 ± 0.3
Dd2-C_0_^119L-131S-259L^‡	144 ± 12^a^	1538 ±110^a^	15 ± 0.5^a^	255 ± 62^d^	221 ± 50^a^	795 ± 235^c^
Dd2-C_1_^131S-140I-259L^‡	223 ± 36^b^	723 ± 141^a^	25 ± 7.2^a^	501 ± 101^a^	225 ± 73^a^	563 ± 203^d^

* IC_50_ values are the mean ± s.e.m. from at least 3 independent experiments in quadruplicate; one-way ANOVA test followed by a Dunnett’s multiple comparison test of mean IC_50_ values of mutants vs. Dd2 parent line, with p < 0.0001 [*^a^*], p < 0.001 [*^b^*], p < 0.01 [*^c^*], p < 0.05 [*^d^*]

‡ parasite lines harboring a mutation in *pfdhodh*

MYX: myxothiazol; ATV: atovaquone.

**Table 5.** Cross-resistance to ETC inhibitors for *Plasmodium* parasites with different resistance genotypes/phenotypes.

drug	in vitro vs *P. falciparum* IC_50_ (nM)*				

	D6	Dd2	Tm90-C2B	Dd2-D_1_^I22L^	D10yDHOD

T111	0.028 ± 0.009	0.045 ± 0.056	5.6 ± 0.88^a^	0.0008 ± 0.0001^a^	>2,500
ATV	0.10 ± 0.03	0.10 ± 0.05	8,256 ± 921^a^	0.21 ± 0.03	>2,500
ELQ-300	3.74 ± 0.56	3.20 ± 0.29	1.73 ± 0.28	224 ± 19^a^	>2,500
CQ	15 ± 1.2	163 ± 18^a^	208 ±15^a^	248 ± 25^a^	62 ± 7.8

* IC_50_ values are the mean ± s.e.m. from at least 3 independent experiments in quadruplicate; one-way ANOVA test of mean IC_50_ values vs. D6 strain, with [^*a*^], p < 0.01.

ATV: atovaquone; CQ: chloroquine.
